# The Effects of Ninjinyoeito on Impaired Spatial Memory and Prefrontal Cortical Synaptic Plasticity through *α*-Amino-3-hydroxy-5-4-isoxazole Propionic Acid Receptor Subunit in a Rat Model with Cerebral Ischemia and *β*-Amyloid Injection

**DOI:** 10.1155/2023/6035589

**Published:** 2023-09-30

**Authors:** Masaki Nagao, Akinobu Hatae, Kazuma Mine, Soichiro Tsutsumi, Hiroya Omori, Marika Hirata, Maaya Arimatsu, Chise Taniguchi, Takuya Watanabe, Kaori Kubota, Shutaro Katsurabayashi, Katsunori Iwasaki

**Affiliations:** ^1^Institute for Aging and Brain Sciences, Fukuoka University, Fukuoka 814-0180, Japan; ^2^Department of Neuropharmacology, Faculty of Pharmaceutical Sciences, Fukuoka University, Fukuoka 814-0180, Japan

## Abstract

Ninjinyoeito (NYT), a traditional Japanese medicine, is effective for improving physical strength and treating fatigue and anorexia. Recently, a clinical report revealed that NYT ameliorates cognitive dysfunction in Alzheimer's disease (AD) patients, although the mechanisms remain unclear. AD is a neurodegenerative disorder accompanied by a progressive deficit in memory. Current therapeutic agents are largely ineffective in treating cognitive dysfunction in AD patients. In this study, we investigated the effects of NYT on spatial memory impairment in a rat model of dementia. Rats were prepared with transient cerebral ischemia and intraventricular injection of *β*-amyloid_1-42_ for 7 days (CI + A*β*). NYT was orally administered for 7 days after cerebral ischemia. We evaluated spatial memory using the Morris water maze and investigated the expression of *α*-amino-3-hydroxy-5-4-isoxazole propionic acid receptor subunits, the phosphorylation level of glutamate receptor A (GluA)1 at serine sites S831 and S845, and the Ca^2+^/calmodulin-dependent protein kinase II (CaMKII) in the hippocampus and prefrontal cortex of CI + A*β* rats. In the CI + A*β* rats, NYT treatment shortened the extended time to reach the platform. However, NYT did not restore the decrease in the hippocampal GluA1, GluA2, or CaMKII expression but increased prefrontal cortical phosphorylation levels of S845-GluA1 and CaMKII. Therefore, NYT may alleviate spatial memory impairment by promoting glutamatergic transmission involved in the phosphorylation of S845-GluA1 and CaMKII in the prefrontal cortex of CI + A*β* rats. Our results suggest that NYT is a valuable treatment for AD patients.

## 1. Introduction

Alzheimer's disease (AD) is a neurodegenerative disorder and is the most common pathological cause of dementia in older adults [1]. AD is caused by amyloid plaques and neurofibrillary tangles in the brain [2, 3], and the progression of cognitive dysfunction is induced by neuronal loss and atrophy in the hippocampus and cortex [4]. However, current therapeutic agents for dementia cannot completely ameliorate cognitive dysfunction. Ninjinyoeito (NYT) is a traditional Japanese medicine that consists of 12 herbal medicines: ginseng, Japanese angelica root, peony root, rehmannia root, Atractylodes Rhizome, poria sclerotium, cinnamon bark, astragalus root, *Citrus unshiu* peel, polygala root, schisandra fruit, and *Glycyrrhiza*. NYT is used to treat fatigue, anorexia, and declining physical strength in older adults [5]. A clinical report has shown that NYT ameliorates cognitive dysfunction in AD patients [6, 7]. Therefore, NYT may be effective in treating cognitive dysfunction in patients with AD. However, the therapeutic mechanisms of NYT remain unclear.

Glutamatergic signals play a vital role in cognitive functions. Ionotropic glutamate receptors are classified into N-methyl-D-aspartate receptor (NMDA) and *α*-amino-3-hydroxy-5-methyl-4-isoxazole propionic acid (AMPA) receptors. AMPA receptors play an essential role in promoting learning and memory function and are involved in synaptic plasticity, which relies on the efficient synaptic transmission of glutamatergic acids [8]. AMPA receptors comprise four subunits of glutamate receptor A (GluA)1–4 [9], and the changes in the number [10], subunit composition [11], and phosphorylation state of these receptors [12] regulate the efficiency of glutamatergic transmission, which includes long-term potentiation (LTP), a crucial process that facilitates synaptic transmission in synaptic plasticity [13]. GluA1-deficient mice show spatial memory impairment [14], and the loss of GluA2 and GluA3 has been observed in the entorhinal cortex and hippocampus of patients with AD [15].

In addition, Ca^2+^/calmodulin-dependent protein kinase II (CaMKII), which is a multifunctional protein kinase that specifically promotes autophosphorylation [16], plays a crucial role in synaptic plasticity and memory function [17]. Autophosphorylation of CaMKII is associated with the elevation of Ca^2+^ influx through the NMDA receptor, which leads to LTP [18, 19]. The brains of AD patients [20] and *β*-amyloid (A*β*)_1-42_-treated hippocampal neurons [21] show decreased phosphorylated CaMKII in the dendrites and synapses of hippocampal neurons. Therefore, these observations demonstrate that these factors, such as the activity of AMPA receptors and CaMKII, are relevant to glutamatergic transmission efficiency and are involved in the cognitive dysfunction of AD.

We previously developed a dementia rat model prepared with transient cerebral ischemia and intraventricular injection of A*β*_1-42_ (CI + A*β*) [22] that exhibits spatial memory impairment and apoptotic neuronal death in the hippocampus [23]. Our previous studies showed that spatial memory impairment in CI + A*β* rats improves with the administration of donepezil (DPZ), an acetylcholinesterase inhibitor [22], and traditional natural medicines [23]. In this study, we investigated the effects of NYT on spatial memory impairment and the expression of AMPA subunits and phosphorylated CaMKII in CI + A*β *rats.

## 2. Materials and Methods

### 2.1. Animals

Rats were kept in a temperature-controlled room (23°C ± 2°C), with a relative humidity of 60% ± 5% and a 12-h light/dark cycle. Food and water were available ad libitum. All animal care and use procedures were carried out in accordance with the regulations dictated by the Experimental Animal Care and Use Committee of Fukuoka University (#2009053).

### 2.2. Rat Dementia Model with the CI + A*β* Operation

Ten-week-old male Wistar rats (weighing 300–350 g) were obtained from Kyudo Co., Ltd. (Saga, Japan). A*β*_1-42_ peptides were purchased from AnaSpec Inc. (Fremont, CA, United States). Rats were anesthetized intraperitoneally using three different mixtures of anesthetics and immobilized in a stereotaxic instrument. Anesthetics were prepared with 0.375 mg/kg medetomidine (Meiji Seika Pharma Co., Ltd., Tokyo, Japan), 2 mg/kg midazolam (SANDOZ K.K., Holzkirchen, Germany), and 2.5 mg/kg butorphanol (Meiji Seika Pharma Co., Ltd.). Cerebral ischemia and intracerebroventricular injection of A*β*_1-42_ (AnaSpec Inc., CA, United States) were performed according to our previous study [23]. In brief, the bilateral vertebral arteries were permanently cauterized using electrocautery. The bilateral common carotid arteries were threaded from the back of the vessels for easy temporary occlusion. Immediately after cerebral ischemia, the aggregated A*β*_1-42_ (600 pmol/20 *µ*l) was injected bilaterally daily for 7 days using a perfusion pump (CMA/100; Microdialysis AB, Stockholm, Sweden). Sham-operated control rats were prepared by threading the common carotid arteries without occlusion.

### 2.3. Morris Water Maze Task

The Morris water maze (MWM) task was modified according to Morris [24] and was identical to that described previously [25]. A circular swimming pool (diameter 150 cm, height 45 cm; Neuroscience Inc., Tokyo, Japan) was filled with clear water with a temperature of 23°C ± 2°C to 2 cm above the surface of the transparent platform. The platform (diameter 12 cm, height 30 cm) was placed in the center of one quadrant of the pool. Each rat underwent acquisition training, which consisted of three trials daily for 5 consecutive days. Each rat was placed into the water at one of the three starting quadrants that did not contain the platform. Rats that reached the platform within 30 s on the 5th trial day were deemed to have memorized the maze and subsequently underwent the CI + A*β* operation the next day. In the probe test, rats were allowed to swim freely for 180 s without the platform. Their performance in the probe test was assessed by the latency to pass the platform position for the first time. The results of all measures were recorded on a personal computer for behavioral analysis using Axis-30 (Neuroscience Inc.).

### 2.4. Western Blotting

Rats were sacrificed immediately after the probe test. The hippocampus and prefrontal cortex were dissected, isolated, and homogenized using a T-PER Tissue Protein Extraction Reagent (Thermo Fisher Scientific, MA, USA), which contained a protease inhibitor cocktail (Nacalai Tesque, Inc. Kyoto, Japan) and a phosphatase inhibitor cocktail (Nacalai Tesque, Inc.), and centrifuged (4°C, 20400 × *g* for 30 min) to extract the proteins. Protein concentrations were measured using a BCA Protein Assay Reagent Kit (Thermo Fisher Scientific). Proteins were separated using sodium dodecyl sulfate-polyacrylamide gel electrophoresis (Bio-Rad, Hercules, CA, USA) and transferred to polyvinylidene fluoride membranes. The membranes were blocked with Blocking One (Nacalai Tesque, Inc.) in distilled water, followed by incubation overnight at 4°C with specific primary antibodies: anti-GluA1 antibody (1 : 1000, 182003, Synaptic Systems, Gottingen, Germany), anti-GluA2 antibody (1 : 1000, MAB397, Merck, Darmstadt, Germany), anti-GluA3 antibody (1 : 1000, MAB5413, Merck), anti-phospho-GluA1 S831 antibody (1 : 1000, ab109464, Abcam, Cambridge, UK), phospho-GluA1 S845 antibody (1 : 1000, ab76321, Abcam), anti-CaMKII (1 : 000, 4436, Cell Signaling Technology, Massachusetts, USA), anti-phospho-CaMKII (1 : 1000, 12716, Cell Signaling), anti-caspase-3 (1 : 1000, 9665S, Cell Signaling), and anti-*β*-actin antibody (1 : 1000, ab8227, Abcam). The membranes were then incubated with horseradish peroxidase-conjugated secondary antibody (1 : 10000, NA934, GE Healthcare, CA, USA) for 1 hour at room temperature. Immunoreactive polypeptides were detected by chemiluminescence using ImmunoStar Zeta or LD (Wako, Osaka, Japan). The mean ratios of S831 and S845 of the phosphorylation were estimated by comparing total GluA1 expressions. The phosphorylation of CaMKII to CaMKII ratio was calculated by dividing the phosphorylation of CAMLII by the total CaMKII expression.

### 2.5. Drug and Treatment Schedule

NYT (lot: 2190108020, 352223500, and 372176700) dried powder extract was provided by Tsumura & Co. (Tokyo, Japan) and was produced from 12 species of crude drugs: Angelicae acutilobae radix (4.0 g, root of *Angelica acutiloba* Kitagawa), poria (4.0 g, fungus of *Poria cocos* Wolf), Rehmanniae radix (4.0 g, root of *Rehmannia glutinosa* Liboschitz), Atractylodis rhizoma (4.0 g, root of *Atractylodes japonica* Koidzumi), Ginseng radix (3.0 g, root of *Panax ginseng* C.A. Meyer), Cinnamomi cortex (2.5 g, bark of *Cinnamomum cassia* Blume), Aurantii nobilis pericarpium (2.0 g, peel of *Citrus unshiu* Markovich), Polygalae radix (2.0 g, root of *Polygala tenuifolia* Willdenow), Paeoniae radix (2.0 g, root of *Paeonia lactiflora* Pallas), Astragali radix (1.5 g, root of *Astragalus membranaceus* Bunge), Glycyrrhizae radix (1.0 g, root of *Glycyrrhiza uralensis* Fisher), and Schisandrae fructus (1.0 g, fruit of *Schisandra chinensis* Baillon). The ingredients of NYT were demonstrated by the three-dimensional high-performance liquid chromatography (HPLC) profile, which is provided by Tsumura & Co. (Figure S1).

Rats were divided into three groups: a sham-operated group, a CI + A*β* vehicle-treated group, and a CI + A*β* NYT-treated group. NYT was dissolved in distilled water. NYT at doses of 1000 and 2000 mg/kg was orally administered after A*β* injection once per day for 7 days. It has been reported that NYT at a dose of 1000 mg/kg was effective on the scopolamine-induced impairment of passive avoidance response and apathy-like behavior in mice [26, 27], and NYT 1500 mg/kg recovered the reduced motivation of nest-building behavior in the water immersed mice [28]. The pharmacokinetics of NYT at 2000 mg/kg was also revealed in the brains of mice [29]. Therefore, we selected the dose of NYT at 1000 and 2000 mg/kg in this study. The sham and CI + A*β* vehicle-treated groups were orally administered distilled water following the same schedule. The final administration of drugs was performed 1 hour before the probe test of the MWM task. Rats were divided into 4 groups: sham-operated group, CI + A*β* vehicle-treated group, CI + A*β* NYT 1000 mg/kg-treated group, and CI + A*β* NYT 2000 mg/kg-treated group.

### 2.6. Statistical Analysis

All data were analyzed using one-way analysis of variance, followed by post hoc Dunnett's tests, using Prism 7 (GraphPad Software, CA, USA). The criterion for statistical significance was set at *p* < 0.05. Values are expressed as means ± standard errors of the mean. One outlier was removed from the vehicle group data for the prefrontal cortical expression of p-GluA1 (S845) using robust regression, followed by the outlier identification method (Prism 7).

## 3. Results

### 3.1. Effect of NYT on Spatial Memory Impairment

**Figure 1:**
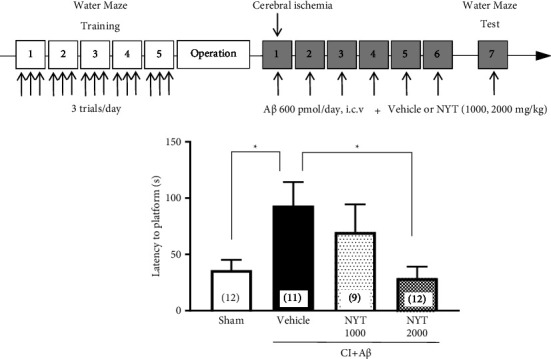
The effect of Ninjinyoeito (NYT) on spatial memory impairment in a rat model with cerebral ischemia and beta-amyloid injection (CI + A*β*). Spatial memory in rats was evaluated as the initial time taken to pass through the platform area during the Morris water maze. Bar graphs represent the sham-operated group (*n* = 12), CI + A*β* vehicle-treated group (*n* = 11), CI + A*β* NYT 1000 mg/kg-treated group (*n* = 9), and CI + A*β* NYT 2000 mg/kg-treated group (*n* = 12). ^*∗*^*p* < 0.05 vs. the CI + A*β* vehicle group. Data were analyzed using the one-way analysis of variance, followed by Dunnett's tests.

We first examined the effect of NYT on spatial memory impairment in CI + A*β* rats. Spatial memory was evaluated by the latency to pass the platform position of the water maze. The CI + A*β* rats had longer latencies than the sham rats (Figure 1, *p* < 0.05), which indicated that the CI + A*β* rats had a spatial memory impairment. Treatment of CI + A*β* rats with NYT at a dose of 1000 mg/kg did not change the extended time to reach the platform; however, NYT at a dose of 2000 mg/kg significantly shortened the extended time to reach the platform (Figure 1, *p* < 0.05). Therefore, NYT treatment significantly ameliorated spatial memory impairment in CI + A*β *rats.

### 3.2. Effect of NYT on the Expression of AMPA Receptor Subunits and the Phosphorylation Level of GluA1 in the Hippocampus

**Figure 2:**
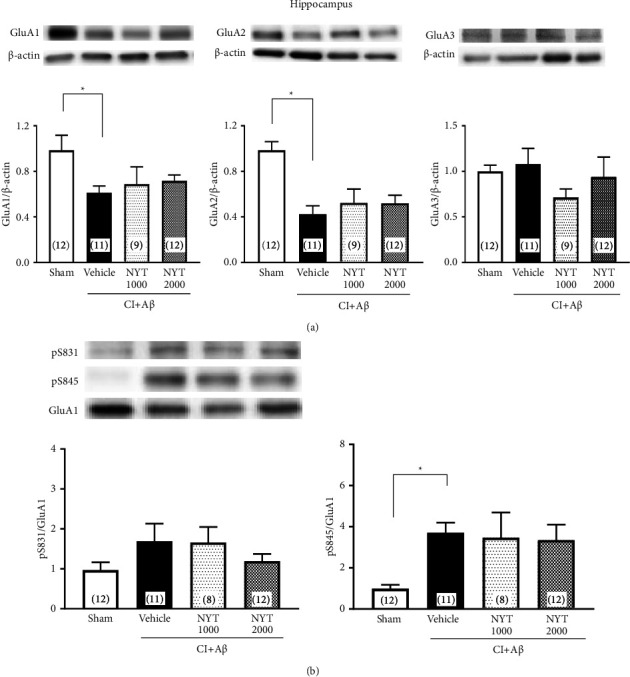
Effect of Ninjinyoeito (NYT) on the expression of AMPA subunits and the phosphorylation level of GluA1 in the hippocampus of CI + A*β* rats. Quantitative western blotting analysis shows the expression of GluA1, GluA2, and GluA3 in the hippocampus of the CI + A*β* rats (a). Representative western blots of the phosphorylation of GluA1 at serine sites S831 and S845 and total GluA1. Phosphorylation levels at S831 and S845 were estimated in the hippocampus of CI + A*β* rats (b). Bar graphs represent the sham-operated group (*n* = 12), CI + A*β* vehicle-treated group (*n* = 11), CI + A*β* NYT 1000 mg/kg-treated group (*n* = 8–9), and CI + A*β* NYT 2000 mg/kg-treated group (*n* = 12). ^*∗*^*p* < 0.05 vs. the CI + A*β* vehicle group. Data were analyzed using one-way analyses of variance, followed by Dunnett's tests.

Since the CI + A*β* rats previously showed spatial memory impairment and apoptotic neuronal death in the hippocampus [23], we first studied the mechanisms of NYT in the hippocampus. The majority of subunits in the hippocampal neurons of adult rats are GluA1/2 and GluA2/3 [30–32]; however, GluA4 is not involved in the signal transmissions that mediate the AMPA receptors in the hippocampus pyramidal neurons [33, 34]. Therefore, we assessed the protein expression of hippocampal GluA1–3. The CI + A*β* operation significantly decreased the expression of GluA1 and GluA2 in the hippocampus (Figure 2(a), *p* < 0.05). However, NYT treatment did not restore this decreased expression in the hippocampus (Figure 2(a)). There was no change in GluA3 expression in the hippocampus (Figure 2(a)). We then investigated the effect of NYT on the phosphorylation level of GluA1 at the S831 and S845 sites, which mediate the AMPA receptor trafficking [35–37], in the hippocampus of CI + A*β* rats. The GluA1 phosphorylation level at S831 did not change in the hippocampus of CI + A*β* rats (Figure 2(b)). In contrast, the hippocampal S845 phosphorylate level of GluA1 increased following the CI + A*β* operation; however, NYT did not affect this increase (Figure 2(b), *p* < 0.05). Therefore, NYT did not have a beneficial effect on the change in AMPA subunits in the hippocampus of CI + A*β *rats.

### 3.3. Effect of NYT on the Expression of AMPA Subunits and the Phosphorylation Level of GluA1 in the Prefrontal Cortex

**Figure 3:**
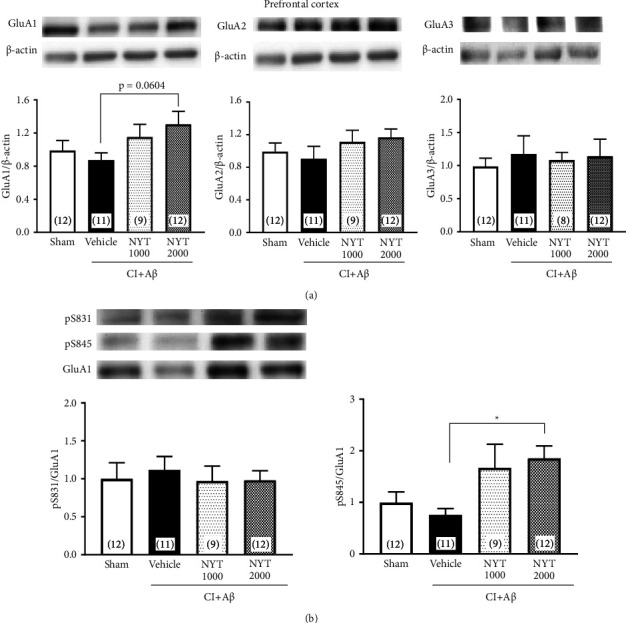
Effect of Ninjinyoeito (NYT) on the expression of AMPA subunits and the phosphorylation level of GluA1 in the prefrontal cortex of CI + A*β* rats. Quantitative western blotting analysis shows the expression of GluA1, GluA2, and GluA3 in the prefrontal cortex of CI + A*β* rats (a). Representative western blots of the phosphorylation of GluA1 at serine sites S831 and S845 and total GluA1. The phosphorylation levels at S831 and S845 were estimated in the prefrontal cortex of CI + A*β* rats (b). Bar graphs represent the sham-operated group (*n* = 12), CI + A*β* vehicle-treated group (*n* = 11), CI + A*β* NYT 1000 mg/kg-treated group (*n* = 8–9), and CI + A*β* NYT 2000 mg/kg-treated group (*n* = 12). ^*∗*^*p* < 0.05 vs. the CI + A*β* vehicle group. Data were analyzed using the one-way analysis of variance, followed by Dunnett's tests.

Since the synaptic plasticity of the spatial memory function in the MWM task has previously been induced in the prefrontal cortex [38], we focused on the prefrontal cortex in CI + A*β* rats. The CI + A*β* rats did not exhibit any changes in the expression of GluA1 and GluA2 in the prefrontal cortex (Figure 3(a), *p* < 0.05). However, NYT treatment showed a trend increase in GluA1 expression in the prefrontal cortex of CI + A*β* rats (Figure 3(a), *p*=0.0604). There was also no change in the GluA3 expression in the prefrontal cortex (Figure 3(a)). For the phosphorylation level of GluA1, although no difference was observed between the CI + A*β* and sham rats, NYT treatment significantly increased the phosphorylation level of S845 in the CI + A*β* rats (Figure 3(b), *p* < 0.05). Therefore, NYT promoted AMPA receptor signaling via the phosphorylation of GluA1 at S845 in the prefrontal cortex of CI + A*β *rats.

### 3.4. Effect of NYT on the Phosphorylation Level of CaMKII in CI + A*β* Rats

**Figure 4:**
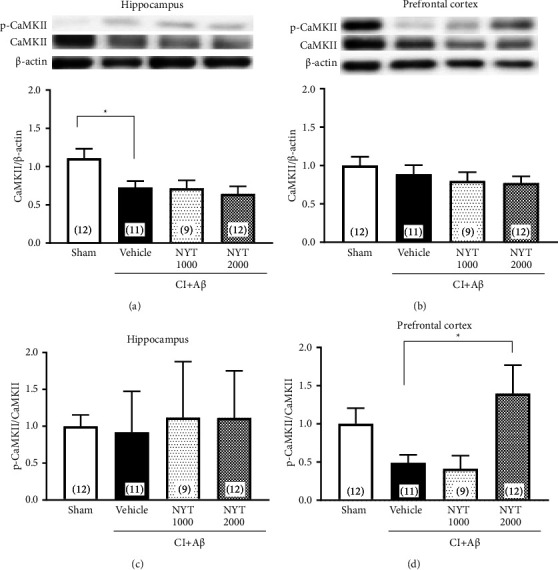
Effect of Ninjinyoeito (NYT) on the phosphorylation level of CaMKII in the CI + A*β* rats. Quantitative western blotting analysis of the phosphorylation ratio of phosphorylated Ca^2+^/calmodulin-dependent protein kinase II (p-CaMKII) and total CaMKII in the hippocampus (a, c) and prefrontal cortex (b, d) of CI + A*β* rats. Bar graphs represent the sham-operated group (*n* = 12), CI + A*β* vehicle-treated group (*n* = 11), CI + A*β* NYT 1000 mg/kg-treated group (*n* = 9), and CI + A*β* NYT 2000 mg/kg-treated group (*n* = 12). ^*∗*^*p* < 0.05 vs. the CI + A*β* vehicle group. Data were analyzed using the one-way analysis of variance, followed by Dunnett's tests.

We then investigated the effects of NYT on the total protein expression and phosphorylation ratio of CaMKII in the hippocampus and prefrontal cortex of CI + A*β* rats. The total CaMKII expression decreased in the hippocampus of CI + A*β* rats; however, NYT treatment did not restore this expression (Figure 4(a), *p* < 0.05). There was no change in the total expression of CaMKII in the prefrontal cortex of CI + A*β* rats (Figure 4(b)). Although the phosphorylation level of CaMKII did not show significant differences in the hippocampus or prefrontal cortex between sham and CI + A*β* rats, NYT treatment significantly increased the phosphorylation level in the prefrontal cortex of CI + A*β* rats (Figures 4(c) and 4(d), *p* < 0.05). Therefore, NYT enhanced the activation of CaMKII in the prefrontal cortex of CI + A*β *rats.

## 4. Discussion

We investigated the effects and mechanisms of NYT on spatial memory impairment in CI + A*β* rats. We found that NYT treatment restored the prolongation of the latency to reach the MWM platform induced by the CI + A*β* operation to the same degree as the latency to reach the MWM platform in sham rats (Figure 1), which suggests that NYT effectively alleviates the spatial memory impairment in CI + A*β* rats. A previous study reported that NYT ameliorates memory impairment in mice induced by scopolamine using the passive avoidance test [26]. Furthermore, a clinical study by Kudou et al. found that in patients with AD, combined treatment with NYT and DPZ provides a greater improvement in cognitive impairment than treatment with DPZ alone [6]. Similarly, Ohsawa et al. reported that NYT treatment ameliorates cognitive dysfunction in AD patients [7]. Our results of NYT treatment for spatial memory impairment in CI + A*β* rats are consistent with previous studies.

The CI + A*β* rats showed a decrease in the expression of GluA1 and GluA2 in the hippocampus, whereas the expression of GluA3 did not change (Figure 2(a)). Somme et al. reported that the expression of GluA1 and GluA2 decreases in the CA1 hippocampal subfield of rats following transient cerebral ischemia, whereas GluA3 expression does not change [39]. We previously demonstrated the hippocampal apoptotic neuronal death and spatial memory impairment in CI + A*β* rats [23], and similar results were observed in the present study. The CI + A*β* rats showed an increase in caspase-3 expression in the hippocampus, but not the prefrontal cortex (Figures S2(a) and S2(b)). Given that the GluA2 subunit is impermeable to Ca^2+^ and plays a role in preventing neuronal cell death induced by cerebral ischemia [40], the decreased GluA2 expression was likely caused by the apoptotic neuronal death in the hippocampus of CI + A*β* rats. We found that NYT treatment did not restore the expression of hippocampal GluA1 and GluA2 (Figure 2(a)), which may be because NYT does not prevent apoptotic neuronal death in the hippocampus of CI + A*β* rats (Figure S2(a)).

**Figure 5:**
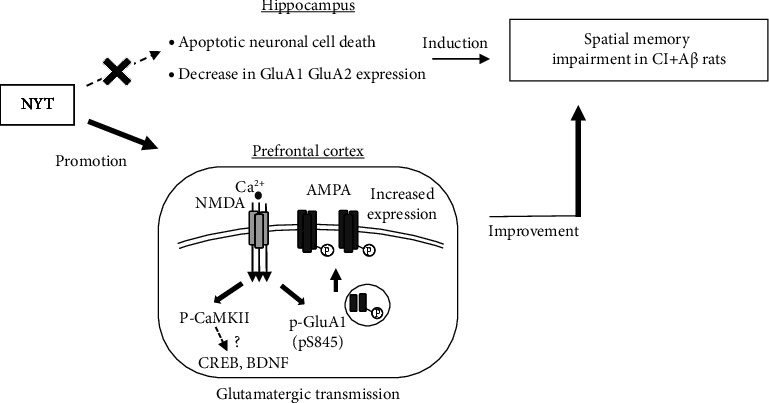
Schematic diagram summarizing the effect of NYT on impaired spatial memory in the CI + A*β* rats. Our result suggested that spatial memory impairment was induced by the decreased expression of GluA1 and GluA2 and apoptotic neuronal death in the hippocampus of CI + A*β* rats. NYT alleviated spatial memory impairment by enhancing the phosphorylation level of GluA1 at serine site S845 or CaMKII in the prefrontal cortex of CI + A*β *rats.

In the prefrontal cortex, the expression of AMPA subunits did not significantly differ between the CI + A*β* and sham rats. In contrast, NYT treatment showed a trend increase in GluA1 expression, but not GluA2 and GluA3 expression, in CI + A*β* rats (Figure 3(a)). Moreover, NYT treatment significantly increased the phosphorylation level of GluA1 at S845, but not GluA1 at S831, in the prefrontal cortex of CI + A*β* rats (Figure 3(b)). These results may have occurred because there was no apoptotic neuronal death in the prefrontal cortex of CI + A*β* rats (Figure S2(b)). It is well established that changes in the number and phosphorylation state of GluA1 regulate glutamatergic transmission efficiency [10, 12]. A highly stimulated glutamate AMPA receptor induces Ca^2+^ inflow via the glutamate NMDA receptor, which results in the phosphorylation and activation of phosphoenzymes, such as CaMKII and protein kinase A (PKA). The activated CaMKII and PKA phosphorylate the S831 and S845 of GluA1, respectively [19, 41], which subsequently leads to the trafficking of AMPA receptors containing GluA1 to the synaptic surface [42]. These signals accelerate the depolarization of NMDA receptors and glutamatergic transmission. Moreover, it has been reported that the phosphorylation of S831 increases channel conductance [43], whereas the phosphorylation of S845 increases the opening probability of AMPA receptors [44]. Hao et al. reported that in AD mice, phosphorylated GluA1 ameliorates spatial memory impairment, as assessed by the MWM task [37], and Xue et al. also reported that amphetamine increases S845, but not S831, phosphorylation, in the striatum and medial prefrontal cortex of rats [45]. Therefore, our findings suggest that NYT alleviates spatial memory impairment in CI + A*β* rats by promoting the prefrontal cortical phosphorylation of S845 of GluA1 and in turn, synaptic plasticity (Figure 5). The phosphorylation level of CaMKII did not show a significant difference in the prefrontal cortex between sham and CI + A*β* rats; however, there seemed to be a tendency of decrease in the level (Figure 4(d)). Thus, the changed CaMKII signaling in the prefrontal cortex may be related to the spatial memory impairment of CI + A*β* rats. By investigating the downstream of the CaMKII signaling pathway, the detailed effects of NYT on memory impairment would be clear in our further studies. The phosphorylated CaMKII and CaMKIV trigger the phosphorylation of the transcription factor cyclic-adenosine monophosphate response element-binding protein [46, 47] and promote brain-derived neurotrophic factor (BDNF) transcription [48]. BDNF is highly expressed in the hippocampus and cortex [49] and plays a vital role in the modulation of neuronal development [48] and the induction of long-lasting LTP [50]. Therefore, the increased phosphorylation level of CaMKII induced by the NYT treatment may be involved in synaptic plasticity by promoting the BDNF pathway in the prefrontal cortex of CI + A*β* rats (Figure 5).

NYT consists of 12 herbal medicines, and it has been reported that Japanese angelica root and *Citrus unshiu* peel contained in NYT are effective for treating cognitive dysfunction. Our previous study revealed that Japanese angelica root alleviates spatial memory impairment in a rat model of cerebral ischemia [51]. In addition, in cerebral ischemic rats, nobiletin extracted from *Citrus unshiu* peel ameliorates memory deficits and stimulates the phosphorylation of CaMKII, which enhances LTP [52]. Therefore, the effect of NYT on spatial memory impairment in CI + A*β* rats may be specifically related to these herbal medicines.

## 5. Conclusions

We found that NYT is effective in alleviating spatial memory impairment in CI + A*β* rats. Our findings suggest that NYT promotes glutamatergic transmission involved in the expression and phosphorylation of GluA1 and increases the phosphorylation level of CaMKII in the prefrontal cortex of CI + A*β* rats. Therefore, NYT treatment may be a valuable alternative therapeutic agent to treat cognitive dysfunction in patients with AD.

## Data Availability

The data that support the findings of this study are available from the corresponding author upon request.

## Supplementary Materials

Supplementary Materials Figure S1: the three-dimension HPLC profile of the ingredients of NYT. Figure S2: quantitative western blot analysis of the expression of caspase-3 in the hippocampus and prefrontal cortex of CI + A*β *rats.
