# Supplementary material for: The Effects of Ninjinyoeito on Impaired Spatial Memory and Prefrontal Cortical Synaptic Plasticity through α-Amino-3-hydroxy-5-4-isoxazole Propionic Acid Receptor Subunit in a Rat Model with Cerebral Ischemia and β-Amyloid Injection
Source: Evid Based Complement Alternat Med. 2023 Sep 30;2023:6035589. doi: 10.1155/2023/6035589 (PMC10560115; doi:10.1155/2023/6035589)
Supplement: Supplementary Materials — Figure S1: the three-dimension HPLC profile of the ingredients of NYT. Figure S2: quantitative western blot analysis of the expression of caspase-3 in the hippocampus and prefrontal cortex of CI + Aβ rats. [file 6035589.f1.docx]

**Supplementary Materials**


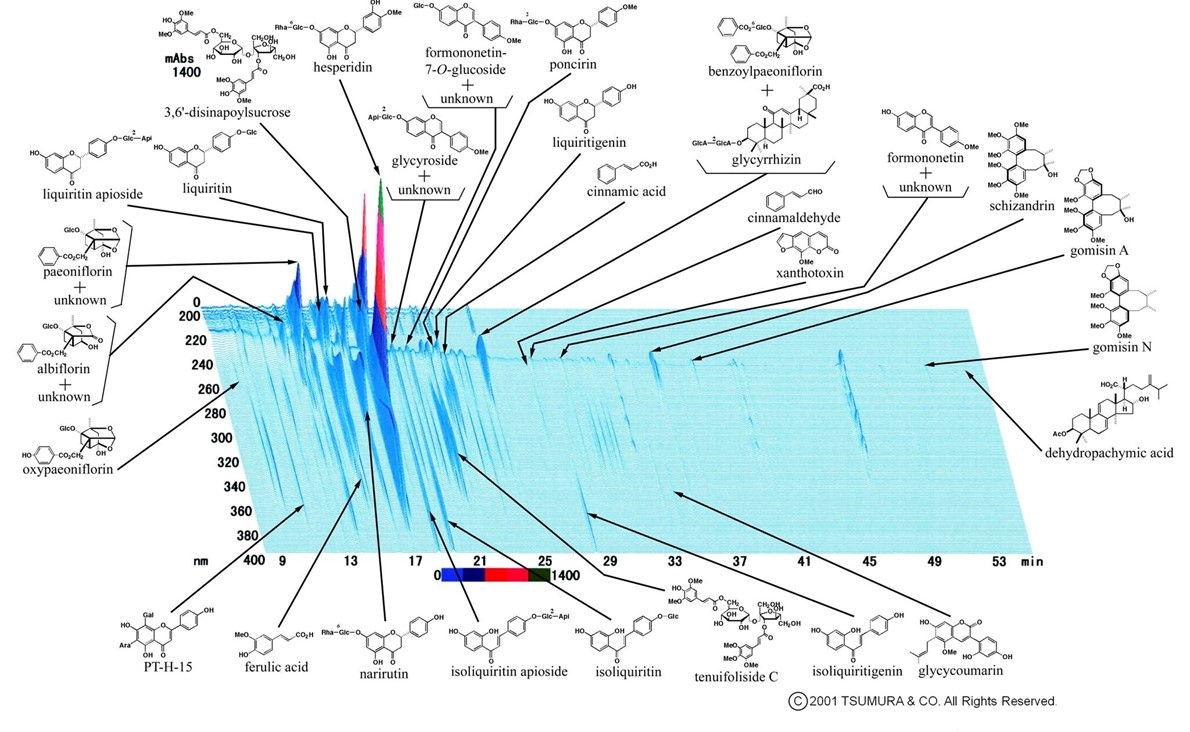


Supplementary figure 1.

The ingredients of NYT are shown by the three-dimension high-performance liquid chromatography (HPLC) profile. The 1.0 g dried extract of NYT was extracted with 20 mL methanol by ultrasonication for 30 min and was centrifuged at 3000 rpm for 5 min. The supernatants were filtered through a 0.45 µm membrane filter, and then submitted for HPLC analysis. HPLC apparatus consisted of a Shimadzu LC 10A (analysis system software: CLASS-M10A ver. 1.64, Tokyo, Japan), a multiple wavelength detector with a range of UV 200-400 nm (Shimadzu SPD-M10Avp, diode array detector), an auto injector (Shimadzu CTO-10AC) and a column; ODS (TSK-GEL 80TS, 250 × 4.6 mm i.d., TOSOH, Tokyo, Japan, Column temperature: 40℃). A gradient elution was employed with solvent A (0.05M phosphate ammonium, pH 3.6) and solvent B (100% acetonitrile). The ratio of solvent B was changed from 10% to 100% linearly over 60 min with a flow rate at 1.0 mL/min. In the analysis, the maximum detection sensitivity of NYT was set at 1400 milli-absorbance (mAbs).


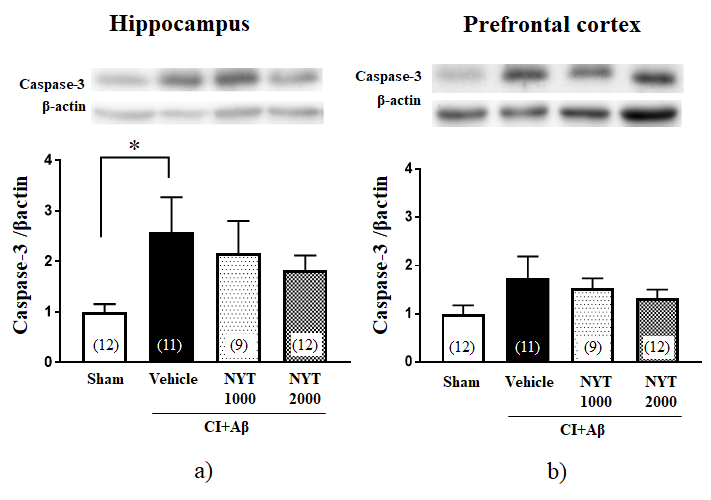


Supplementary figure 2

Quantitative western blotting analysis of the expression of caspase-3, which is a primary mediator of cell apoptosis, in the hippocampus (a) and prefrontal cortex (b) of CI+Aβ rats. Bar graphs represent the sham-operated group (n = 12), CI+Aβ vehicle-treated group (n = 11), CI+Aβ NYT 1000 mg/kg-treated group (n = 9), and CI+Aβ NYT 2000 mg/kg-treated group (n = 12). **p* < 0.05 vs. the CI+Aβ vehicle group. Data were analyzed using one-way analyses of variance, followed by Dunnett’s tests.
